# Validating an Agency-based Tool for Measuring Women’s Empowerment in a Complex Public Health Trial in Rural Nepal

**DOI:** 10.1080/19452829.2016.1251403

**Published:** 2016-11-08

**Authors:** Lu Gram, Joanna Morrison, Neha Sharma, Bhim Shrestha, Dharma Manandhar, Anthony Costello, Naomi Saville, Jolene Skordis-Worrall

**Affiliations:** ^a^ Institute for Global Health, University College London, London, UK; ^b^ London School of Hygiene and Tropical Medicine, London, UK; ^c^ Mother Infant Research Activities, Kathmandu, Nepal; ^d^ World Health Organization, Geneva, Switzerland

**Keywords:** Capability approach, Gender, Human development, Human rights, Measurement

## Abstract

Despite the rising popularity of indicators of women’s empowerment in global development programmes, little work has been done on the validity of existing measures of such a complex concept. We present a mixed methods validation of the use of the Relative Autonomy Index for measuring Amartya Sen’s notion of agency freedom in rural Nepal. Analysis of think-aloud interviews (*n* = 7) indicated adequate respondent understanding of questionnaire items, but multiple problems of interpretation including difficulties with the four-point Likert scale, questionnaire item ambiguity and difficulties with translation. Exploratory Factor Analysis of a calibration sample (*n* = 511) suggested two positively correlated factors (*r* = 0.64) loading on internally and externally motivated behaviour. Both factors increased with decreasing education and decision-making power on large expenditures and food preparation. Confirmatory Factor Analysis on a validation sample (*n* = 509) revealed good fit (Root Mean Square Error of Approximation 0.05–0.08, Comparative Fit Index 0.91–0.99). In conclusion, we caution against uncritical use of agency-based quantification of women’s empowerment. While qualitative and quantitative analysis revealed overall satisfactory construct and content validity, the positive correlation between external and internal motivations suggests the existence of adaptive preferences. High scores on internally motivated behaviour may reflect internalized oppression rather than agency freedom.

## 1. Introduction

Women’s empowerment is a central policy issue on the international development agenda and the subject of numerous international commitments including the Sustainable Development Goals (UN General Assembly 2015), the Beijing Platform for Action (UNWOMEN 1995) and the Cairo Declaration on Population and Development (UNPD 1994). In addition to its intrinsic value (Sen 1999), women’s empowerment has been associated with improved child health (Cunningham et al. 2014; Malapit et al., “Women’s Empowerment in Agriculture,” 2013), fertility outcomes (Jejeebhoy 1995; Upadhyay et al. 2014) and health service utilization (Ahmed et al. 2010). Empowerment has variously been defined as an “expansion in people’s ability to make strategic life choices in a context where this was previously denied them” (Kabeer 1999) and “the expansion of agency” (Ibrahim and Alkire 2007, 437). Although a complex concept, it has a central focus on the availability of options, the freedom to make choices and the power to influence and control (Malhotra and Schuler 2005).

Quantitative indicators of women’s empowerment have become increasingly popular in global development policy (Harper et al. 2014; Kishor and Subaiya 2008; Malhotra and Schuler 2005; UN 2014). The majority of such indicators rely on proxy indicators such as education, employment and political representation of women (UN 2014) or individual control over assets (Doss 2013) or direct measures such as intra-household decision-making and freedom of movement (Kishor and Subaiya 2008). Individual authors have devised their own tools for measuring empowerment (Shroff et al. 2009).

Several studies have highlighted the insufficiency of existing measures. Proxy measures do not allow us to cleanly separate the causes or effects of empowerment from empowerment itself (Agarwala and Lynch 2006). For example, we would not be able to tell, using the proxy measure of education, whether women’s education causes or is caused by greater empowerment among women. Proxy measures may also be inaccurate representations of empowerment, as studies have shown inconsistently varying correlations between direct measures of empowerment and proxy measures across contexts (Vaz, Pratley, and Alkire 2016). In the policy context, the Millennium Development Goal targets were criticized for narrowly substituting educational attainment for a broad-based conception of empowerment (Kabeer 2005; North 2010; Sen and Mukherjee 2014).

As regards, direct measures of empowerment, intra-household decision-making is a complex process of negotiation, delegation and agenda-setting (Acharya and Bennett 1983; Agarwal 1997; Kabeer 2001; Mumtaz and Salway 2009), which is not well captured by a single reductive question on who the key decision-makers are. It is often biased by the social desirability of a male “breadwinner” (Allendorf 2007; Kabeer 1997; Mumtaz and Salway 2009; Vogler and Pahl 1994) and comparative studies have found low agreement rates between responses given by husbands and wives in both high-income (Davis 1971) and low-income settings (Allendorf 2007; Godoy et al. 2006; Jejeebhoy 2002) on questions regarding decision-making authority.

Independent mobility may also be undesirable for women where male or female accompaniment is a sensible means of protection from harassment or assault. Social norms may also confer praise, prestige and family support on women who conform to seclusion norms. In such contexts, women may autonomously choose to limit their own mobility out of self-interest and unaccompanied mobility may be a sign of poverty and lack of social capital rather than independence (Kabeer 1999, 2001; Mumtaz and Salway 2005).

Researchers have begun to use the Relative Autonomy Index derived from Self-Determination Theory (SDT) (Deci and Ryan 2000) as a direct measure of women’s empowerment (Ibrahim and Alkire 2007). In addition to being studied independently (Vaz, Pratley, and Alkire 2016), it also forms part of the widely used Women’s Empowerment in Agriculture Index (Alkire et al. 2013; Malapit et al., “Measuring Progress Toward Empowerment,” 2013; Sraboni et al. 2014). By using the framework of SDT to capture Amartya Sen’s notion of “agency freedom” (Sen 1985) as autonomously motivated behaviour, this tool potentially avoids the pitfalls of existing measures of empowerment by focussing on local women’s own experience of freedom (Ibrahim and Alkire 2007) instead of externally imposed measures of decision-making power and independent mobility.

Although the Relative Autonomy Index was proposed for use as a standardized and internationally comparable tool (Ibrahim and Alkire 2007), little published work exists to examine the hypothesis that it is indeed valid for use across sites in low-income contexts. We cannot assume that this is the case, as the more conventional indicators based on decision-making or mobility have been found to reflect empowerment in one setting while simultaneously being irrelevant or reflecting a lack of empowerment in another setting (Mason 1986). To our knowledge only one published study from Chad (Vaz, Pratley, and Alkire 2016) and one unpublished study from Bangladesh (Vaz et al. 2013) have reported on the measurement properties of the Relative Autonomy Index in low-income contexts. Hence, it remains an important empirical question whether a contextually sensitive construct such as women’s empowerment can or should be measured using the Relative Autonomy Index across multiple contexts, as persistent concerns regarding the context sensitivity of measures of empowerment have undermined the scope for comparative research in the past (Agarwala and Lynch 2006).

Our study contributes to the evidence gap on the context sensitivity of measures of women’s empowerment through a validation study of the Relative Autonomy Index in rural Nepal.

## 2. Conceptual Review of “Empowerment” and “Agency”

Despite extensive debate and reconceptualization of empowerment in the development literature, no single, clear definition of empowerment has emerged (Trommlerová, Klasen, and Leßmann 2015). Similar to analytical and normative understandings of power (Haugaard 2010), some of this confusion may stem from a conflation of empowerment as a good to be promoted and empowerment as an analytical phenomenon. In analytical usage, we are studying causal or correlational relationships and in fact observe a great deal of overlap among the concepts of “freedom,” “power” and “empowerment” in the literature. Theoretical writings on empowerment frequently refer to both expanding “freedoms” and gaining “powers” (Ibrahim and Alkire 2007; Kabeer 1999; Mosedale 2005) and indicators of empowerment typically include both decision-making “powers” and “freedom” of movement (Kishor and Subaiya 2008; Malhotra and Schuler 2005). Debates over the distinction between power and freedom admit substantial conceptual overlap (Morriss 2009) while literature on power and freedom develop parallel concepts.^1^ Consequently, we will draw on all three concepts in the following sections.

This study follows Ibrahim and Alkire (2007) in understanding empowerment as an expansion of human “agency.” Agency is integral to the Capability Approach of Amartya Sen who defines agency as “what the person is free to do and achieve in pursuit of whatever goals or values he or she regards as important” (Sen 1985, 203). However, Sen’s own definition of agency is potentially undertheorized in important ways: First, Sen’s notion of “agency freedom” is committed to understanding agency from an individualistic perspective. His definition specifically refers to what a “person” is free to do as opposed to what a “group of persons” is free to do. Development practitioners often need to measure and analyse the collective power dynamics of groups, e.g., in the design and planning of participatory community-development programmes (Hickey and Mohan 2004) and it is worth noting that the collective freedom of groups differs markedly from the freedom of individuals (Cohen 1983; Ibrahim 2006; Pelenc et al. 2013; Peterson and Zimmerman 2004; Stewart 2005). Suppose a poor mother can only afford one bowl of rice for herself and her child. Both may be individually free to eat the entire bowl as neither mother nor child has any desire to actively prevent the other from doing so. However, collectively the mother and child are unfree to have a bowl each.^2^

Secondly, Sen’s definition is silent on whether agency should be located internally or externally. For individuals, agency could be viewed as internal cognitive, emotional powers or as powers in external interpersonal relationships. Sen may have meant to include both aspects in his definition of agency, as they are likely linked, but in practice the external aspect is measured more frequently (Kishor and Subaiya 2008; Malhotra and Schuler 2005). In this paper, we have chosen to locate agency in interpersonal relationships.

We could operate with a conception that does not emphasize the psychosocial domain at all, but sees agency as the ability to achieve any end by any means, e.g., in the concept of “power-to” (Allen 1998). However, such a wide denotational range of “agency” would allow virtually any improvement in standard development outcomes to constitute empowerment and potentially conflate empowerment with the entirety of the development enterprise. Studies using measures such as education, household assets, health or employment status (Vyas and Watts 2009) often risk falling into this group, since women are considered empowered if any of these outcomes improve regardless of whether their psychosocial conditions substantially change.

Finally, Sen’s definition is silent on the role of status-relationships in defining agency. There are three ways we might conceptualize the role of status-relationships. First, in Rowland’s (1997) concept of “power-with,” empowerment is rooted in mutually supportive status-relationships.^3^ For example, the third phase of the SASA! Intervention to reduce domestic violence in Uganda was described as “joining ‘power with’ others to support change” (Kyegombe et al. 2014). In this view, social capital (Coleman 1988) or social support (Gottlieb and Bergen 2010) could be seen as constitutive of agency by enabling women to better draw on the support of others to achieve their valued aims and goals.

Second, Rowland’s (1997) concept of power as “power-over” another person sees conflict and struggle as intrinsic to the empowerment process. Here, greater equality or even superiority in hierarchical status-relationships is seen as constitutive of agency. This view coincides with views of power (Dahl 1957) and freedom (Berlin 1959) (“negative freedom”), which sees agency as the ability to pressure others to change or resist pressures from others to change in turn. Measures of empowerment such as decision-making power (Acharya et al. 2010; Allendorf 2007; Shroff et al. 2009) fall into this group as women are often classified as empowered the more often they participate in decisions in the household or are sole decision-makers.

Finally, we may not consider mutually supportive, egalitarian or hierarchical status-relationships as empowering in themselves, but rather emphasize the importance of legitimacy in status-relationships. An empowered individual is not necessarily entirely free from external influences, but only free from unwanted, illegitimate external influences (Hirschmann 2009; Pettit 1996). Individuals may autonomously choose to defer to others in domains where they have no interest in making decisions or where they consider others better informed. We see this orientation in measures of “ideal decision-making power” (Peterman et al. 2015) where respondents are scored on whether they believe the people who actually have decision-making power in the household are also the people who should have decision-making power. This view comes closest to the measure of agency that we employ in this paper.

Drawing on the preceding discussion, we can now provide a more precise definition of agency for the purposes of this study. We define agency as “the ability of individuals to pursue and achieve valued goals free of illegitimate restraints posed by personal relationships and with the help of legitimate support from personal relationships.” It specifically excludes measuring the collective agency of groups, agency of individuals to overcome internal, mental barriers and agency understood as the ability to achieve any goal in any way. It also excludes views of agency as intrinsically concerned with garnering support or with overcoming power struggles. “Empowerment” in turn is defined as the expansion of agency (Ibrahim and Alkire 2007).

Our definition of agency aligns most closely with a measurement tool proposed by Deci and Ryan (2000) in Ibrahim and Alkire (2007) called the Relative Autonomy Index.^4^ The tool measures agency based on the degree to which individuals endorse their behaviour as willingly enacted in accordance with their authentic interests and values. Motivations for behaviour are seen as falling on a continuum from the fully self-determined to the fully external. Derived from SDT, four levels of progressively increasing autonomy are distinguished (Deci and Ryan 2000):

- *External regulation*—Behaviour is controlled by specific external contingencies and people behave to attain desired consequences due to tangible rewards or to avoid punishment.
- *Introjected regulation*—Control of behaviour comes from contingent consequences administered by individuals to themselves. Prototypical examples are pride, guilt and shame.
- *Identified regulation*—The underlying value of a behaviour is recognized and accepted as a means to an end rather than a source of intrinsic enjoyment and satisfaction. Examples include exercising solely for the purpose of improving one’s health.
- *Integrated regulation*—Behaviours have been fully accepted and brought in harmony with other aspects of a person’s values and identity.

While external and introjected regulation is considered external types of motivation, identified and integrated regulation are considered internal types of motivation. A fifth type of motivation standing outside the classification scheme above is called “amotivation” and reflects a general lack of intention to act. Amotivated people either do not act at all or simply “go through the motions” without reflection (Deci and Ryan 2000). Individuals reporting higher levels of internal and lower levels of external motivation or amotivation are seen as possessing greater self-determination, agency and empowerment.

The survey tool used in this study was designed to reflect a variety of motivations by asking women to agree or disagree with reasons offered for their behaviour in the domains of work outside the household, household chores, health seeking^5^ and group participation on a four-point Likert scale. For example, participants were asked if they perform their household chores because they have to, because they find them valuable or because they will get into trouble if they do not do them.

## 3. Methods

### 3.1. Context

Nepal is among the poorest countries in the world with 25.2% of the population living below the national poverty line (World Bank 2015). In all, 22–27% of its GDP comes from remittances and 75% of the population is employed in agriculture (CIA 2014). Nepal has traditionally been a conservative, religious society with 81% of the population self-identifying as Hindus, 9% as Buddhists and 4% as Muslims. Local interpretations of Hinduism prescribe caste- and gender-based social hierarchies based on notions of purity and pollution (Bennett 1983). Having recently emerged from a violent civil war, the country is still listed by the UK Department for International Development as a fragile state.

This study was embedded within a four-arm cluster randomized controlled trial. The trial was being conducted in 60 village development committee clusters of Dhanusha and Mahottari district. These districts form part of the Central Terai in Nepal, where the local language is Maithili. Clusters were randomly allocated to receive either (1) Participatory women’s groups alone (2) Women’s groups combined with cash transfers (3) Women’s groups combined with food transfers (4) Control. Details of the trial have been reported elsewhere (Saville et al., forthcoming). Women’s groups had previously proven effective in reducing maternal and neonatal deaths (Prost et al. 2013), but their impact on pregnancy nutrition and low birth weight was as yet unknown. Researchers had hypothesized that women’s groups achieved their impact on health outcomes through women’s empowerment (Rosato et al. 2008; Victora and Barros 2013). The data for this study were collected at baseline before the start of the trial intervention in order to inform the development of a tool to measure women’s empowerment for the impact evaluation phase of the trial.

### 3.2. Methodological Approach

We used mixed methods to check for: (1) content validity to ensure consistency between researcher and respondent understanding of questionnaire items (Bowden et al. 2002), (2) reliability to ensure that the instrument is free of gross random error (Lohr 2002) and (3) construct validity to verify that the instrument enables the researcher to make sound inferences (Seppälä et al. 2009). Content validity was explored through think-aloud interviews and discussions with field staff. Reliability was assessed using spot checks of previously filled questionnaires and calculations of inter-rater variability in empowerment scores. Construct validity was assessed using factor analysis and comparison of empowerment scores across a priori predictive categories of empowerment.

**Figure 1.**
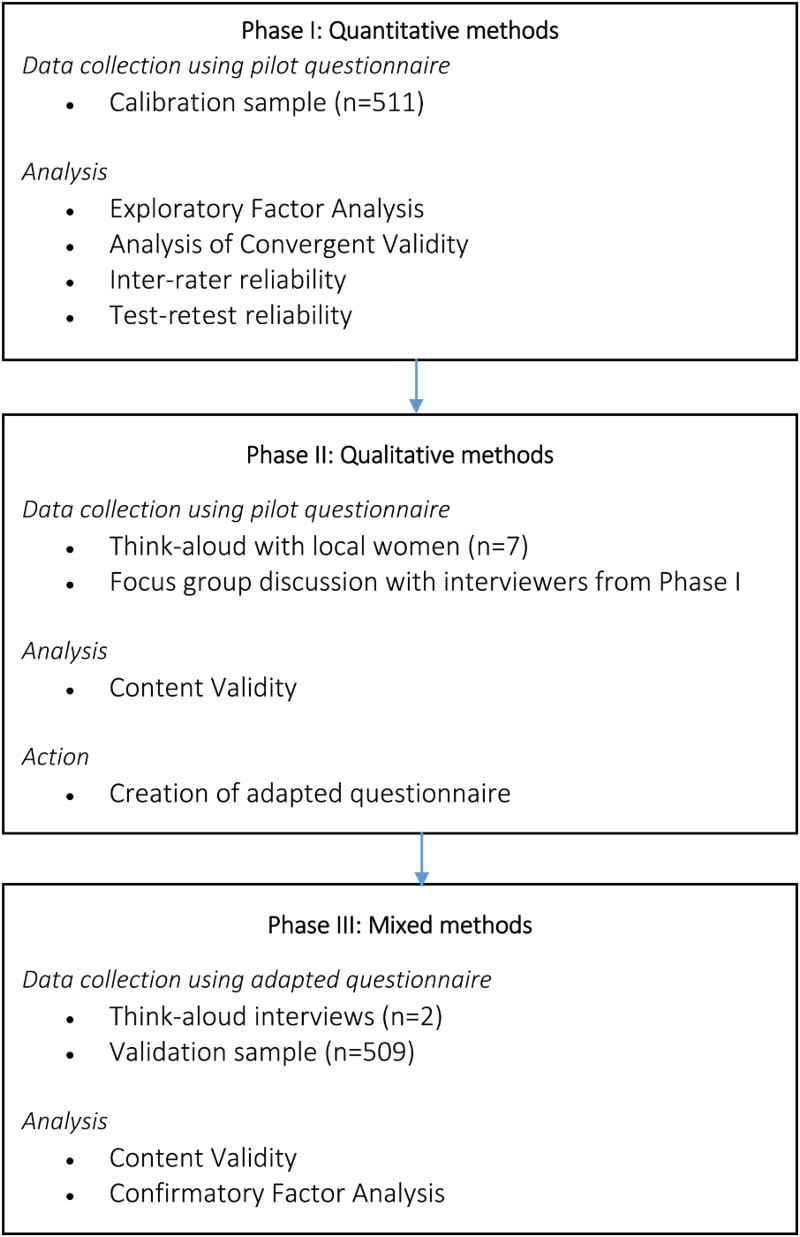
Flow of quantitative and qualitative data collection.

The data were collected and analysed in three phases (Figure 1). First, a pilot questionnaire was administered and construct validity of the survey data was assessed using exploratory factor analysis (EFA) and convergent validity analysis between October and December 2013. Reliability of the survey data were assessed using measures of inter-rater variability and test–rest reliability. Secondly, between January and February 2014, qualitative think-aloud interviews were conducted with local women, and a focus group discussion (FGD) was held with interviewers from the first stage of surveying. We analysed the results of this qualitative round to explore content validity and modified the pilot questionnaire to create the adapted version of the questionnaire. Finally, we conducted further think-aloud interviews and collected quantitative data in May 2014 using the newly adapted tool. We performed Confirmatory Factor Analysis (CFA) to verify construct validity and qualitative analysis to verify content validity.

Our approach allowed us to use both qualitative and quantitative methodologies to explore content and construct validity, which cannot be adequately assessed with one method alone (Johnson, Onwuegbuzie, and Turner 2007). While statistical methods may be able to tell us if the tool behaves numerically as expected, they cannot tell us if the items in the tool are perceived as reflecting intrinsic or extrinsic motivation in the minds of the respondents. Nor can they tell us why respondents may have difficulty providing answers to certain questionnaire items or how the questionnaire should be modified. Conversely, qualitative methods are limited in their sampling and cannot inform us about the population distribution of responses or whether respondent answers are correlated in expected or unexpected ways.

### 3.3. Qualitative Data Collection and Analysis

#### 3.3.1. Think-aloud interviews

Our main approach to assessing the content validity of the tool was through think-aloud interviews, an interviewing methodology derived from social and cognitive psychology (Collins 2003). In traditional “rehearsal” piloting methods, interviewers run through questionnaires with a participant while an observer notes down length, flow, salience and ease of administration. In think-aloud interviews, the interviewer asks the respondent how they went about answering the survey question. Think-aloud interviews allow researchers to explore whether respondents understand the concept behind a question or are simply providing answers out of politeness. They complement statistical techniques by exploring not only whether the survey tool captures the underlying concept or not, but why it would not capture that concept and what changes would improve its cognitive-conceptual fit.

#### 3.3.2. Sample

In January 2014, Lu Gram (LG) observed seven think-aloud interviews by Neha Sharma (NS) and assistant Kabita Sah (KS) on respondents purposefully selected from the trial surveillance system to reflect a range of socio-economic strata. Four interviews were digitally recorded and translated directly into English by NS and KS. Three participants refused to be recorded so NS made notes summarizing each statement, and translated these into English. Data were collected in Maithili and Nepali, the native language of NS and KS. One FGD was conducted with the fieldwork team who had collected data using the original pilot questionnaire to identify problematic questions and triangulate our findings. In March 2014, the pilot questionnaire was shortened and modified and NS conducted two additional think-aloud interviews with the revised questionnaire (Table 1).

**Table 2.** Baseline characteristics of participants in calibration (*N* = 511) and validation (*N* = 509) samples

Address	Sample		Where would you go if you had simple health problems?	Sample	
	Calibration	Validation		Calibration	Validation
Husband’s family home	488 (96%)	493 (97%)	Public sector health institution	77 (15%)	92 (18%)
Respondent’s natal home	21 (4%)	16 (3%)	Private medical centre, clinic, nursing home or hospital	89 (17%)	71 (14%)
Other	2 (0.4%)	0 (0%)	Pharmacy/medical shop	294 (58%)	308 (61%)
			Other specify	19 (4%)	13 (3%)
**Head of household in Husband's home**			Don’t do anything	29 (6%)	19 (4%)
Husband	129 (25%)	162 (32%)	Don’t know	3 (1%)	6 (1%)
Father-in-law	213 (42%)	210 (41%)	* *		
Mother-in-law	67 (13%)	57 (11%)	**Do you voluntarily participate in any group?**		
Other man from husband’s household	9 (2%)	4 (1%)	Yes	34 (7%)	53 (10%)
Other woman from husband’s household	6 (1%)	4 (1%)	No	477 (93%)	456 (90%)
Woman herself	87 (17%)	56 (11%)	* *		
Unknown	0 (0%)	16 (3%)	**Literacy**		
			Cannot read	320 (63%)	343 (67%)
**Husband working outside Nepal**			Reads with difficulty	114 (22%)	71 (14%)
No	252 (49%)	273 (54%)	Reads easily	77 (15%)	95 (19%)
Yes	259 (51%)	236 (46%)	* *		
**Age of woman (years)**			**Which type of work are you involved in?**		
≤18	51 (10%)	33 (6%)	Only work inside the household	314 (62%)	283 (56%)
19–24	182 (36%)	196 (39%)	Work outside the home	6 (1%)	14 (3%)
25–34	223 (44%)	209 (41%)	Both	191 (37%)	212 (42%)
35+	55 (11%)	71 (14%)

**Table 1.** Summary of participants in qualitative data collection

Respondent no.	Age	Socio-economic status	Education	Religion	Audio recording available	Questionnaire version
1	20	Low	Illiterate	Hindu	Yes	Original
2	20–30	High	Unknown	Hindu	No	Original
3	40	Low	Illiterate	Hindu	No	Original
4	25–30	Medium	Illiterate	Hindu	Yes	Original
5	20	Low	Quranic school up to 5th level	Muslim	Yes	Original
6	20	Low	Could read with difficulty	Muslim	Yes	Original
7	45	High	Studied up to 10th standard	Hindu	No	Original
8	20	Low	School Leaving Certificate (SLC)	Hindu	Yes	Revised
9	25	Medium	Studied up to 5th standard	Hindu	Yes	Revised

#### 3.3.3. Analysis

LG read the transcripts and filled a two-dimensional table, labelling rows by respondent and domain of application and columns by motivation types. A summary of every respondents’ general understanding of each motivation was generated and illustrative quotes were extracted. Observation field notes informed this process by marking out points of tension, lack of comprehension or disturbance by other family members during interviews. After analysing the think-aloud data, results were fed back and discussed with the local field team to check the consistency of our findings with their experiences.

### 3.4. Quantitative Data Collection and Analysis

#### 3.4.1. Exploratory factor analysis

We used EFA to study the fit between the empirical behaviour of our indicators and the structure of agency proposed by SDT.^6^ EFA is a statistical method for uncovering the underlying structure of a set of indicators. By choosing the statistical model that best models correlations between the indicators, it allows the analyst to summarize the interrelationships between a large set of variables in terms of a smaller set of dimensions called “factors” (Thompson 2004). A number of approaches exist to assessing the fit of a particular factor structure including the number of factors: use of a scree plot (Cattell 1966), the Kaiser criterion (Kaiser 1960) and use of fit statistics such as Root Mean Square Error of Approximation (RMSEA), Comparative Fit Index (CFI), Tucker–Lewis Index (TLI) and the Standardized Root Mean Square Residual (SRMSR) (Hu and Bentler 1999). In the “scree plot” method, we plot the additional variance explained by adding another factor against the total number of factors. The amount of variance accounted for by a factor is called the “eigenvalue” of the factor and the ideal number of factors is selected based on where a “kink” is perceived to exist in the scree plot (Cattell 1966). The Kaiser criterion limits the choice of factors by ruling out factors with eigenvalue greater than 1 (Kaiser 1960). For the RMSEA and the SRMSR, a good fit is indicated by a value less than 0.06, an adequate fit by a value less than 0.08 and a poor fit by a value greater than 0.08. For the CFI and TLI, a good fit is indicated by a value greater than 0.95, an adequate fit by a greater than 0.90 and a poor fit by a value less than 0.90 (Hu and Bentler 1999). It is generally recommended to avoid exclusively relying on statistical indicators of model fit and to consider whether the emergent factor structures are substantively plausible in deciding on the final number of factors (Fabrigar et al. 1999).

#### 3.4.2. Confirmatory factor analysis

Exploratory Factor Analysis seeks to reveal the underlying structure in an exploratory, inductive fashion. CFA seeks to confirm or refute a pre-specified model of factor structure (Thompson 2004) using the fit statistics RMSEA, SRMSR, CFI and TLI with the same cut-off values for adequacy of model fit as in EFA. Since CFA is used to verify pre-existing theory rather than generate new theory, we can use CFA to verify the factor structure hypothesized during EFA, using an independent sample from the same population. The sample on which we performed EFA is called the calibration sample and the sample on which we performed CFA the validation sample.

#### 3.4.3. Sample

Trained local field workers collected a calibration sample of interviews from 511 women between 1October and 1 December 2013, using the pilot questionnaire. Respondents constituted randomly selected women who were enrolled in the trial surveillance. To be eligible for the trial surveillance study, women needed to be aged between 10 and 49, married and neither widowed, divorced nor separated. Women who had family planning surgery or whose husbands had a vasectomy were also excluded. From 25 April to 25 August 2014, field workers also interviewed 509 randomly sampled women satisfying the same eligibility criteria, using the revised questionnaire. This constituted the validation sample. Table 2 displays participant characteristics from the calibration and validation samples. Although the father-in-law was usually the head of the household (41–42%), the respondent herself was household head in 11–17% of the population. 80% of women were between the ages of 19 and 34 and 63–67% of the sample could not read. The rate of participation in any groups was very low (7–10%). Additionally, 15–25% experienced months without enough food for family needs, 46–51% of husbands were working outside of Nepal, 14–16% of women were Muslim, the rest Hindu, and 65–70% had already had two or more pregnancies (data not shown in table).

#### 3.4.4. Scoring

We tested and compared two scoring methods for compiling survey responses into a single index.

First, the results were scored using a fixed scoring scheme. This employed a weighted sum of Likert scale scores. Statements reflecting external regulation were scored −4 points for level of agreement, whereas statements indicating introjected regulation scored −2 per level of agreement. Statements reflecting identified or integrated regulation scored +3 points per level of agreement. Finally, an “amotivation” option was scored 0 points indicating inability to make choices due to force of circumstances rather than external coercion. Categorization of statements into motivation types followed the original Chad survey. The approach is the same as the one taken by Vaz, Pratley, and Alkire (2016).^7^

Second, we created scores based on the factor analysis model that emerged in our calibration sample. We created a CFA model based on the results of our EFA by retaining factor components with loadings of magnitude greater than 0.05 and predicted maximum a posteriori factor scores for the underlying latent factors. We predicted factor scores for each domain of agency separately and summed the scores across domains and normalized them to standard deviation 1 and mean 0. For the purposes of comparability, scores from the fixed scoring scheme were similarly normalized.

#### 3.4.5. Construct validity

Construct validity was tested by inspecting the results of factor analysis and comparing them to the predictions of SDT. We also performed an analysis of convergent validity (Seppälä et al. 2009) by comparing the final score across characteristics expected to vary with empowerment status (age, parity, socio-economic status, education, caste, decision-making power). Strength of evidence was assessed using linear regression adjusted for clustering using Huber robust variance estimators. Internal consistency was checked using Cronbach’s *α* and by performing CFA on the validation sample using the factor structure hypothesized from EFA on the calibration sample.

#### 3.4.6. Reliability

Inter-rater reliability was checked by testing for differences in empowerment scores across field workers using an ANOVA. Test–retest reliability was checked by re-interviewing six purposively selected women from the calibration sample. Four re-interviews checked the household chores and health-seeking domains amongst women with average scores from the fixed scoring scheme and the remaining two checked work outside the home and group participation amongst women with extremely low or extremely high scores from the fixed scoring scheme.

## 4. Results

### 4.1. Factor Analysis

**Figure 2.**
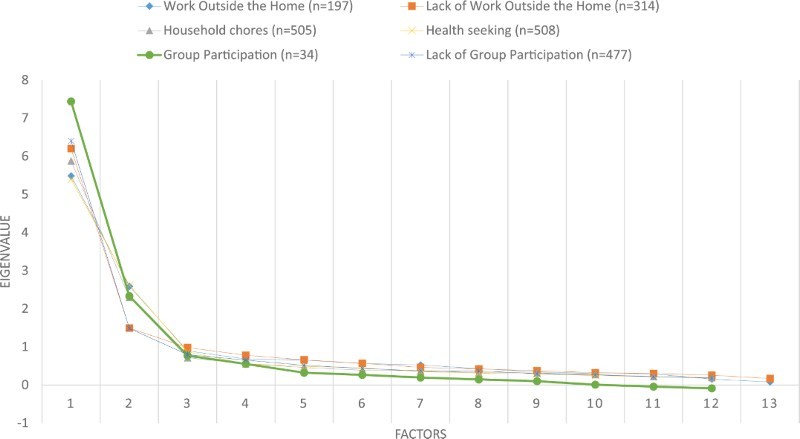
Scree plot of eigenvalues associated with factors.

Figure 2 shows a scree plot of eigenvalues associated with factors for each domain separately. Group participation results should be used with caution due to its small sample size (*n* = 34). Across all domains there was a “kink” at two or three factors. According to the Kaiser criterion (Knott and Bartholomew 1999), two factors was the maximum, as the third eigenvalue was less than one. With respect to fit indices, two factors achieved a good fit or borderline good fit for work outside the home (RMSEA 0.048, TLI 0.98, SRMSR 0.052), household chores (RMSEA 0.050, TLI 0.97, SRMSR 0.037), health seeking (RMSEA 0.077, TLI 0.96, SRMSR 0.043) and group participation (RMSEA 0.091, TLI 0.986, SRMSR 0.067), whereas lack of work outside the home (RMSEA 0.106, TLI 0.89, SRMSR 0.057) and non-group participation (RMSEA 0.126, TLI 0.93, SRMSR 0.42) displayed a poorer fit and only achieved a good fit with three factors (RMSEA and SRMSR < 0.05, TLI > 0.95).

**Figure 3.**
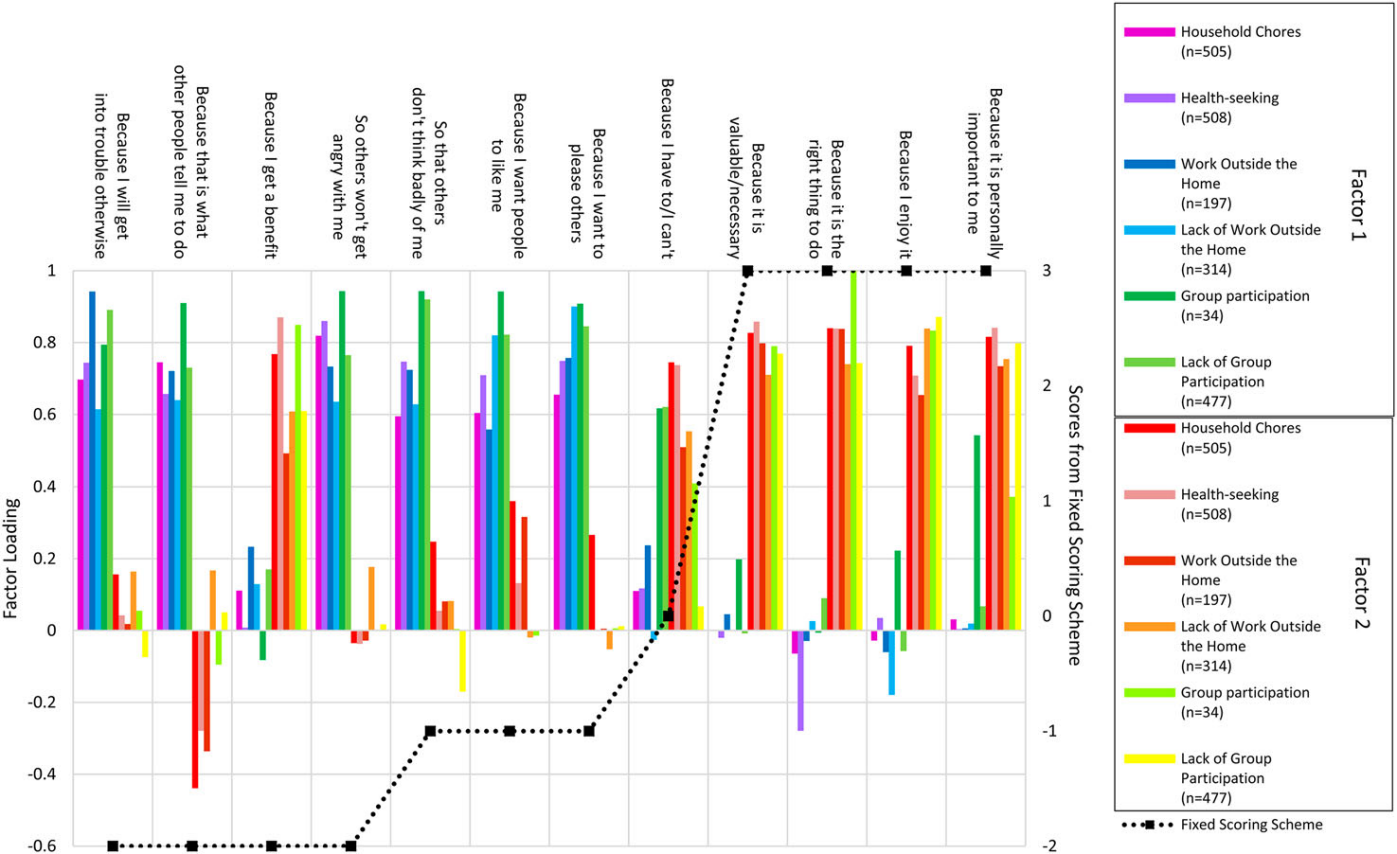
Factor loadings from EFA across domains and motivations.

Figure 3 displays the factor loadings for a two-factor solution across domains as well as the scores from the fixed scoring system. Factors consistently loaded on the same items across all domains except non-group participation, which loaded highly positively on the statement “because I have to” for Factor 2. The two factors were interpretable: Factor 1 related to external pressure and Factor 2 related to internal motivation. The two-factor solution was more stable across domains and had substantially less overlap between factor loadings compared with a three-factor solution, which contained substantial cross-loadings on 7 out of 12 statements (results available upon request). On balance, we opted for the two-factor solution. We generated scores henceforth referred to as Factor E for *external* motivation and Factor I for *internal* motivation by adding factor scores for Factors 1 and 2, respectively, from each domain and normalizing the total to mean 0 and standard deviation 1.

The scores from the fixed scoring system as well as the Factor E and Factor I scores were well approximated by a normal distribution, although ceiling effects were observed in Factors E and I. The two factors are also reflected in crude response rates. For the positively worded domains work outside the home, household chores and health seeking, statements associated with Factors I and E naturally segregated themselves in terms of rates of respondents agreeing with the relevant statements. For Factor I, rates varied from 87% to 97% with an average of 93% and for Factor E, 43–82% with an average of 69%. For the negatively worded domains, non-work outside the home and non-group participation, statements were much more homogeneous. For Factor I, rates varied from 52% to 65% with mean 59% and for Factor E, rates varied from 53% to 70% with mean 60%.

### 4.2. Reliability

In terms of test–retest reliability, the proportion of matching questionnaire items were: 89% for household chores, 79% for health seeking, 54% for work outside the home and 33% for group participation. Caution in interpreting these results is required as the sample size is small and an interval of two months elapsed between survey administration and spot checks.

In terms of inter-observer reliability, the intra-cluster coefficient (ICC) for Factor I was 0.48 (95% CI 0.31–0.66), 0.39 (95% CI 0.23–0.57) for Factor E and 0.20 (95% CI 0.09–0.36) for the scores from the fixed scoring system. This suggests substantial inter-observer variability for Factors I and E, as more than 39% of the variation was attributable to differences between observers, and somewhat lower variability for the scores from the fixed scoring system. The proportion of women interviewed by each field worker reported to agree to all or all but one of the statements varied from 0 to 20% in work outside the home, 0–70% in household chores, 0–82% in health seeking and 0–47% in group participation. The high inter-rater variability is a cause of concern and is expanded upon in Section 5.

### 4.3. Construct Validity

**Figure 4.**
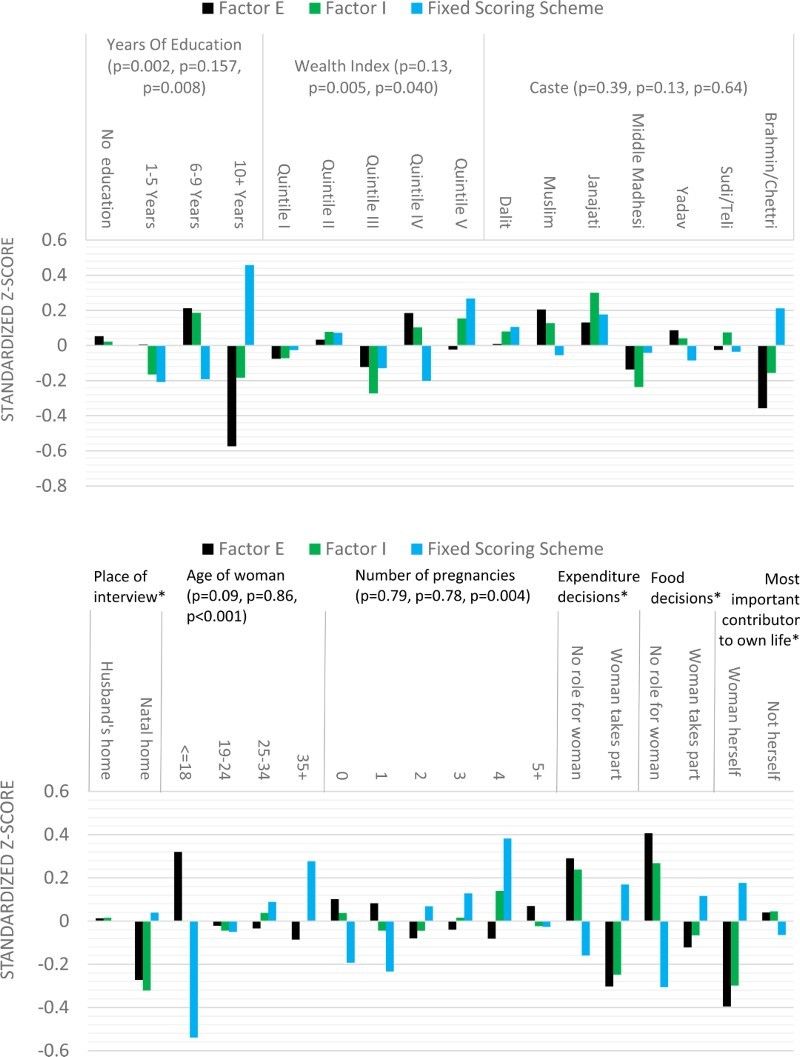
Analysis of convergent validity (*p*-values for a joint effect of the predictor in question on Factor E, Factor I and the scores from a Fixed Scoring Scheme predicted from SDT in brackets). *p-values for the following variables are: place of interview (*p* = 0.17, *p* = 0.22, *p* = 0.87), decisions on expenditures (*p* < .001, *p* < .001, *p* = .007), decisions on food (*p* < .001, *p* = .010, *p* < .001) and most important contributor to own life (*p* = .010, *p* = .02, *p* = .15).

Figure 4 shows the results of a convergent validity analysis. All three scores were observed to vary systematically with predictors. Factor E and the scores from the fixed scoring scheme varied in expected directions. Factor I was often in the opposite direction to that predicted by theory. Highly educated, wealthy, high-caste women who make decisions in the household reported lower levels of Factor E and I and higher scores from the fixed scoring system. Older women, women with greater parity and women staying in their parental home had higher scores from the fixed scoring system and lower levels of Factor E, although there was no evidence for a difference in levels of Factor I (*p* > .22).

In terms of discriminant validity, having a female as opposed to a male interviewer was associated with lower scores from the fixed scoring system (−0.38, *p* = .038) and higher Factor E scores (0.23, *p* = .17), although Factor I scores did not change materially (0.07, *p* = .70). In addition, the presence of other household members at the interview was also correlated with lower scores from the fixed scoring system (−0.33, *p* = .004) and greater Factor E scores (0.37, *p* = .002), while being unrelated to Factor I scores (0.03, *p* = .79).

According to SDT, External Motivation and Internal Motivation are opposites on a continuum (Deci and Ryan 2000). Higher scores from our fixed scoring system, lower Factor E scores and higher Factor I scores were thus theoretically predicted to co-occur. But Factor I moved in the same direction as Factor E across all predictors except the number of pregnancies. The Pearson correlation between Factor E and Factor I was 0.64. Factor correlations in our earlier factor analysis were also positive and ranged from 0.33 to 0.66. The positive correlation between Factor E and Factor I was robust to adjustment for the presence of external observers and the identity of the interviewer with one unit increase in Factor E being associated with 0.59 units increase in Factor I (*p* < .001, 95% CI 0.46–0.72).

### 4.4. Content Validity

Table 3 presents the analysis of the think-aloud data. After an initial reading of the think-aloud data, it became apparent that women’s understanding of questionnaire items did not differ substantially across different domains of application (work outside the home, household chores, health seeking or group participation), as long as the type of motivation reflected by the item remained the same (e.g., “because I have to” vs. “because it is personally important to me”). Hence, results in Table 3 are grouped by motivation rather than by domain.

**Table 3.** Summary of think-aloud data using the original questionnaire (*N* = 7)

Motivation	Narrative description	Illustrative quote(s)
Because it is personally the right thing to do whether or not others agree	Respondents elaborated that they performed their actions of their own initiative, out of self-interest or out of a sense of moral propriety or duty. One respondent even defined moral rightness in terms of obeying one’s guardian.	“I go because of my self-interest whether other people tell me to go or not” - Respondent 3 “Our elderly people used to tell us to go for work whatever people say, to go for work and not to beg food of anyone else. And that is why I completely agree.” - Respondent 6
Because I have to/I can’t	Respondents conveyed a sense of being unconditionally compelled to assume responsibility over work, the household and their children, because nobody else was willing to step in and take over the work as well as fear of scolding by other family members.	“I strongly agree, because I have children at home and so I have to do it to look after them.” - Respondent 4 “I will have to do this work and anyhow nobody will do the work.” - Respondent 7
Because others will get angry otherwise	Participants tended to interpret this question in terms of the need to maintain harmony in the family and avoid conflict, scolding or even physical abuse.	“Listen, if you say something and I also say something then there might be a fight between us. If my guardian scolds me and I don’t say anything back then she won’t fight with me and there will be no conflict between us.” - Respondent 4
Because I want people to like me	Respondents emphasized the ability to garner praise and avoid scolding from family members. While some respondents did not care about non-family members, others enjoyed impressing outsiders.	“Other people will look at me and will say they have visited my home. They will say my home is very clean, my children are very good. When people get together and talk about me, they will say my home is very clean.” - Respondent 6
Because it is personally important to me	Respondents interpreted personal importance in terms of either personal welfare or necessity, the latter meant coping with poverty and taking up work that nobody else was willing to do.	“Yes, it is personally important for me to do this work, because I am the only one to do all this household work at my home.” - Respondent 7
Because I want to please others	There is no translation of the verb “to please” into Nepali/Maithili that preserves the submissive connotations in the English. The closest translation was “Because I want to make others happy”. Respondents tended to agree that they were concerned for other people’s welfare.	“I strongly agree, if I don’t work outside then everybody will be pleased.” - Respondent 5 “We should be happy ourselves and also make other people happy.” - Respondent 4 “Others’ happiness is my happiness.” - Respondent 6
Because I enjoy it	There are two possible Maithili translations for the verb “to enjoy”, *majja* and *nek. Majja* connotes fun, celebration, sensuality, while *nek* connotes general goodness and appreciation. When asked if their work brought them *majja*, respondents vigorously disagreed, emphasizing that they were forced by their poverty to work despite physical and emotional pain. However, when asked if they felt *nek* about their work, respondents readily agreed.	“Since I am ill, but I still have to do this work, how can I enjoy/majja it? … You better write down that I am sick and I have to work! Everyone wants to sit inside and eat food at home just like you people don’t like coming out into the village to ask us questions.” - Respondent 3
Because I will get into trouble otherwise	The Nepali/Matihili translation of this motivation literally means “*Because I will be scolded otherwise*”. Respondents discussed their fear of scolding if they contravened their guardian’s orders or the possibility of praise if they fulfilled their duties well.	“I strongly agree, because people will say good things about me if I do my work.” - Respondent 1 “I strongly disagree. Since my guardian will not permit, then surely I will be scolded if I do work outside.” - Respondent 2
Because others will think badly of me otherwise	Respondents who agreed with this question referred to their fear of scolding and the opportunity for receiving praise from family members. Two respondents did not care for others’ opinion.	“I have to do all of the work, so why won’t I agree with it? I do all of this work, so that I will be praised by everyone and so that my family members will also appreciate me.” - Respondent 6
Because I might get punished otherwise	Respondents unanimously interpreted this question to refer to scolding.	“Because I will be punished if I force my way outside the home. I will be strongly scolded and that is my punishment.” - Respondent 7
Because I might get a benefit	Respondents listed the perceived benefits in response to this question: Ability to feed the family, family happiness and cleanliness, alleviation of health problems, benefits from group membership such as credit and savings.	“I strongly agree, I will be given medicines and if I don’t go to Janakpur hospital for treatment, I will have more severe health problems in the coming days.” - Respondent 1
Because it is valuable	We found no respondents understood the word *arthpurn*. When using *jaruri/*“necessary” respondents understood the question, but this does not capture the aspect of value that goes beyond necessity.	“[jaruri] Yes, this is important. I strongly agree. Actually, it is not important to me. This work could have been done by my daughter or my daughter-in-law. Don’t you think that we should take rest at this stage of life? And because my daughters and my daughter-in-law don’t do this work, I have to do it.” - Respondent 6
Because other people tell me to	When “other people” was interpreted to mean non-family members, respondents tended to disagree and emphasize their own will. When “other people” was interpreted to mean family members, however, respondents tended to agree.	“If you are asking me if other people are telling me to do the work or not, then my neighbours don’t tell me to do any of the work. Do you really think, sister, that my neighbours will tell me to do the work?” - Respondent 6

While there was some divergence in local and intended understandings of some questions, respondents did not struggle with understanding or articulating the motivations behind their actions. However, the statements related to fear of others getting angry with them, punishing them, “getting into trouble,” losing their reputation and the need to be ingratiating were difficult to distinguish from each other. Responses for these reflected a generalized fear of scolding and hope of praise if the respondent conformed to social norms. Field workers and think-aloud respondents informed us of significant complaints regarding the repetitiveness and length of interviews. As a result, we decided to drop the statements “because I might get punished otherwise,” “because I want to please others” and “so that others won’t think badly of me.”

#### 4.4.1. Ambiguous items in the questionnaire

Many respondents remarked that they did not care for the opinions of their neighbours and only listened to their family members.^8^ One respondent who lived alone with her children while her husband worked abroad as foreign labourer repeatedly stated that nobody was there to scold her or punish her. Another respondent stated

[I don’t work outside the home, because people tell me not to] I strongly disagree/asahamat, because it doesn’t matter what other people tell me to do, I will only follow whatever my household members tell me to do.

This is supported by the quantitative data from the calibration sample, where more than 95% of respondents feared punishment from a family member as opposed to a community member, and 75% of respondents felt that a family member would think badly of them if they behaved inappropriately. Social norms in Maithili culture severely restrict mobility outside the household for married women of reproductive age (Acharya and Bennett 1983). Feedback from local employees revealed that casual socializing for young married women with other people in the village was strongly discouraged and it was considered disrespectful to prioritize the opinions of others over those of the family.

It is possible though that the phrasing in the questionnaire might have biased women to thinking about the individual, as opposed to the collective affecting their behaviour. Field workers disagreed with our interpretation and felt that respondents were as likely to be pressured by their family members as by society. In response to whether her household chores were motivated by a desire to be liked by others, one respondent also remarked that she enjoyed impressing visitors with the cleanliness of her home. We decided to ensure removal of any potential ambiguity by changing all references to “other people” to “your family members.”^9^

The statement “because I get a benefit” was interpreted in a variety of domain-specific ways. This ranged from ability to feed her family (work outside the home), family happiness and cleanliness (household chores), alleviation of health problems (health seeking) to group-specific benefits, e.g., loans from credit and savings groups (group participation). According to the fixed scoring scheme from SDT (1), this item is meant to capture only external, disempowering drivers of behaviour such as wage rates. However, in the local context there seemed to be some scope for ambiguity regarding the interpretation of this question. As a result, we dropped this question.

#### 4.4.2. Coping with the Likert scale

It was evident both in body language and speech that respondents struggled with the notion of *agree* vs. *disagree* as well as different shades of agreement. Interviews had to clarify and repeat the words *sahamat*/“agree” and *asahamat*/“disagree” that were used in the questionnaire. Respondents frequently provided clear explanations of their reasoning only to choose a word for “agree” or “disagree” that contradicted what they had told us. For example:

[I do not work outside the home, because I will get into trouble if I do.] I strongly disagree/asahamat with this statement, because my parents will scold me, if I do work outside the home.

*Sahamat* and *asahamat* are primarily Nepali words, but local translations varied from village to village covering phrases such as *raji*/“agree” vs. *nehi raji*/“not agree”, *manjur*/“accept” vs. *nehi manjur*/“do not accept” and *machhin*/“follow” vs. *nehi manchhin*/“do not follow”. This may have contributed to the confusion, although some respondents had not heard of any of the above phrases and only recognized more distantly related adjectives such as *sahi*/“true” vs. *galat*/“false.” Field workers supported this conclusion reporting that respondents’ difficulty with “agree” and “disagree” were major difficulties in conducting interviews. Additionally, asking respondents to additionally distinguish *puri/*“full” agreement from *nehi puri/*“partial” agreement only added to the respondent and interviewer burden. As a result, we decided to reduce the four-point Likert scale to a simple “yes/no” question and change all references to “I” to “you” in the revised questionnaire.

It is possible that the format of the Likert-style questions which required respondents to learn to use a new and unfamiliar classification system at times containing unknown Nepali or Maithili words led respondents to feel as if they needed to “figure out” the “correct answer” to the question rather than give an authentic answer. When the same questions were rephrased into yes/no questions instead of using the Likert scale, respondents were observed to engage with the interviewer more actively by answering in full sentences and explaining her answers instead of answering in monosyllables. As a result, we decided to reduce the four-point Likert scale to a simple “yes/no” question and change all references to “I” to “you” in the revised questionnaire.

#### 4.4.3. English words without exact equivalents in the local language

The statement “Because it is valuable” was difficult to translate. In English, “valuable” connotes utility, monetary value, worthiness and meaningfulness. The direct translation *arthpurn*/“valuable” is an uncommon, intellectual word in the local context. When prompted, no think-aloud respondents had heard the word before and had difficulty understanding it even after explanation, regardless of education or socio-economic background. The team discussed using *jaruri*/“necessary” as a substitute during the pilot phase while recognizing its distinction from “valuable” because some activities have a value in themselves over and beyond necessity. In the end, we decided to drop the motivation “because it is valuable,” because of the difficulty of retaining a faithful translation. We also decided to drop the related item “because it is personally important to me” due to substantial semantic overlap with the item “because it is personally the right thing to do whether or not others agree.” Instead, we added items “because I want to” and “because it is my duty/responsibility,” which were more easily understood statements conveying internal motivation.

For the statement “Because I enjoy it,” we could either use the Maithili phrases *nik lagaai* or *majja lagaai*. While *majja* has connotations of celebration, fun and sensuality, *nik* is much weaker, connoting generalized associations with goodness, likeability and appreciation. Respondents unanimously agreed with statements involving *nik* and disagreed with statements involving *majja*, except one religious group activity, which was described in more sensual terms. They were particularly offended at the mention of *majja* in connection with their work perhaps because it was perceived to trivialize daily sacrifices made for the sake of their families. As a result, we changed the English wording to “Because I like it” to reflect the changes in Maithili.

#### 4.4.4. Adaptations to the original tool

The revised questionnaire incorporated the following changes to the original unadapted tool: (1) The four-point Likert scale was replaced with yes/no questions, (2) References to “I” changed to “you”, (3) References to “other people” changed to “your family members”, (4) Statements that were difficult to translate were dropped, (5) Repetitive statements were dropped and (6) The motivations “because I want to” and “because it is my duty/responsibility” were added. Table 4 shows the final result with a suggested scoring scheme. While scores derived from factor analysis may take local context into better account and can control for measurement error (Agarwala and Lynch 2006), they are always relative to the particular dataset used. A fixed scoring scheme that does not change when the dataset changes would be required for cross-site comparison.^10^

**Table 4.** Final wording of motivations in questionnaire and scoring scheme

Statement	Scoring
*Because you will get into trouble otherwise*	+1 to E if answer is Yes
*Because that is what your family members tell you to do*	+1 to E if answer is Yes
*Because you want your family members to like you*	+1 to E if answer is Yes
*So your family members won’t get angry with you*	+1 to E if answer is Yes
*Because you have to/you can’t*	0
*Because you personally think it is the right thing to do whether or not your family members agree*	+1 to I if answer is Yes
*Because you enjoy it*	+1 to I if answer is Yes
*Because you want to*	+1 to I if answer is Yes
*Because it is your duty/responsibility*	+1 to I if answer is Yes

### 4.5. Construct and Content Validity of the Adapted Tool

New think-aloud data with the revised questionnaire revealed shorter explanation times, improved respondent understanding and a substantially smoother interviewing experience. Importantly, respondents no longer needed repeated probing to provide an answer and did not offer multiple, incompatible interpretations of the same questionnaire item. CFA of the validation dataset revealed adequate fit statistics (RMSEA 0.05–0.08, CFI 0.91–0.99), although the TLI was 0.94–0.99 for the domains of household chores, health seeking and group participation, but only 0.87 for work outside the home indicating poor fit. Factor loadings were high (0.75–0.97) across all four domains except for work outside the home which loaded 0.27 on the motivation “because it is your duty/responsibility” and 0.51 on “because you want to” and group participation which loaded 0.61 on “because it is your duty/responsibility” (see Table 5). This suggested that the motivation “because it is your duty/responsibility” may have been related to a distinct construct from internal or external motivation and should maybe not be used in future tools. Cronbach’s *α* for external and internal motivation was also high (0.79–0.94) indicating good internal consistency.

**Table 5.** Results from CFA on validation sample (*N* = 509)

Factor loadings for each motivation	Work	Household chores	Health seeking	Group participation
**Factor E**				
*Because you will get into trouble otherwise*	0.83	0.85	0.82	0.87
*Because that is what your family members tell you to do*	0.82	0.75	0.86	0.91
*So that your family members won’t get angry with you*	0.94	0.95	0.88	0.94
*Because you want your family members to like you*	0.82	0.89	0.95	0.85
**Factor I**				
*Because you want to*	**0.51**	0.89	0.92	0.94
*Because you personally think it is the right thing to*	0.76	0.82	0.83	0.79
*Because you like it*	0.91	0.87	0.85	0.90
*Because it is your duty/responsibility to do it*	**0.27**	0.97	0.88	**0.61**
RMSEA	0.05	0.08	0.07	0.05
CFI	0.91	0.98	0.99	0.96
TLI	0.87	0.97	0.99	0.94
Cronbach’s *α* for external motivation	0.82	0.82	0.94	0.80
Cronbach’s *α* for internal motivation	0.90	0.79	0.92	0.81

## 5. Discussion

Our results indicate that our simplified and adapted version of the original tool could capture Amartya Sen’s notion of agency freedom. The need for modification of the original tool raises important questions regarding the cross-context sensitivity of measures of women’s empowerment. Agarwala and Lynch (2006) similarly found substantial evidence for cross-cultural variation in their CFA analysis of indicators of women’s decision-making powers and freedom of mobility. They concluded that “it is essential that measures of autonomy remain flexible enough to accommodate contextual changes.”

Factor analysis and think-aloud interviews revealed an ambiguous mixture of interpretations of the motivation “because I have to/can’t” reflecting both force of material circumstances, personal responsibility and coercion by household members (Sections 4.1 and 4.4). This is at odds with our a priori interpretation, in which “because I have to/can’t” reflected “amotivation,” a state of respondents lacking the intention to act altogether. Participants agreeing to the statement “because I have to/can’t” were rather highly motivated, but were motivated by a mixture of internal and external forces.

The motivation “because I get a benefit” also had quite varied interpretations in the think-aloud data such as family happiness and alleviation of health problems (Section 4.4.1). In the EFA, it correlated with indicators of internal motivation (Section 4.1). According to the framework of SDT, the statement “because I get a benefit” reflected primarily externally imposed financial incentives due to research suggesting that financial incentives “crowd out” intrinsic motivation (Deci and Ryan 2000). Differences between the interpretation of this statement in our study and in other studies in high-income settings (Lepper and Greene 2015) may be due to context-specific differences in the meaning ascribed to incentives. Previous research has shown complex effects on intrinsic motivation depending on whether incentives were perceived to acknowledge positive individual traits such as skill and talent or reflect a lack of control over one’s own life (Eisenberger, Pierce, and Cameron 1999; Folbre and Nelson 2000).

These results suggest important ambiguities in cross-cultural understanding of particular questionnaire items. We modified the tool to remove these ambiguities by dropping the “amotivation” items, which were scored zero in any case, and the items with the phrasing “because I get a benefit,” because of their ambiguous interpretation across contexts.

We also found no justification for more severe scores for certain external motivations compared to others, as suggested by the original scoring scheme (Section 4.1). This result was also obtained in the study carried out by Vaz, Pratley, and Alkire (2016) who found that a measure based on only two broad types of motivation may capture the same information as a measure based on three types of motivation. Consequently, in our revised scoring scheme we only include two factors, Factor I for internal motivation and Factor E for external motivation.

The high inter-rater variability indicates a potential limitation of our data (Section 4.2). However, as field workers were dispersed across a wide geographical area, this variation may also reflect genuine cultural diversity across villages. Previous studies in Bangladesh have found substantial variation in empowerment measures explained by village-level fixed effects (Balk 1997; Pitt, Khandker, and Cartwright 2006) and comparably high regional ICCs were also observed in one clinical trial on community mobilization approaches to domestic violence prevention (Abramsky et al. 2014). We performed a standardization exercise of interviewers using the revised tool in August 2015 by rotating 11 interviewers among 12 women four times to estimate inter-rater variability when holding the identity of the respondent constant. We found an ICC of 0.11 for Factor E and 0.00 for Factor I. Caution is advised as the sample size is small, but this suggests that cultural diversity across villages may account for a large part of the ICC observed in our previous data.

Yet, still an ICC of 0.11 still posed a modest risk of bias. Interviewers may have influenced responses subtly through changes in phrasing and intonation, respondents might have responded to interviewer characteristics such sex, age or ethnicity and different respondents may even have consented to participate differently depending on interviewer characteristics (Elliott and West 2015). With potentially sensitive questions concerning women’s agency freedom, interviewers may also have differed in their ability to secure privacy for the respondent or establish trust and rapport. Inadequate standardization and monitoring of field workers cannot be ruled out as sources of this variation. However, the empowerment tool itself may also have been an inherently difficult tool to administer compared to standard epidemiological surveys, which do not require respondents to reflect deeply on human values and freedoms. Indeed, field workers reported that the questionnaire was repetitive and took a long time to complete and they had difficulty understanding and explaining the concepts behind questionnaire items. For this reason, we recommend either controlling for interviewer bias at the design stage by randomizing interviewers to respondents or controlling for bias at the analysis stage by including random or fixed effects based on interviewer identity in the analysis model whenever this is feasible (Elliott and West 2015; Johannes, Crawford, and McKinlay 1997).

Results from linear regressions indicated satisfactory construct validity (Section 4.3). Direct validation came from the lower pressure experienced by women who participated in household decisions. We would not expect a perfect correlation between agency freedom and ability to participate in household decisions, as the two measures are measuring distinct theoretical construct. However, it would not be unreasonable to expect women who participate in household decisions on money and food to be more likely to experience greater agency freedom. Similarly, we would expect partial overlap between agency freedom and belief in own ability to change life, which is what was found in the data. Thus the partial correlations found reinforce the construct validity of our measure.

Our results indicated that women experienced less external pressure when they were interviewed in their natal home (Section 4.3). They also experienced less pressure when they were older than 18 or had at least one child. This is consistent with widely accepted notions of the female life cycle in Nepal, Bangladesh, Pakistan and North India (Acharya and Bennett 1983; Bennett 1983; Davis 2009; Mandelbaum 1993; Minturn and Kapoor 1993). In all, 88% of women in our sample were married between ages 12 and 18. The group of women falling in the age group 18 years or less would likely be considered *kanya*s or “newly married” in Maithili culture. Traditionally, *kanya*s would be under the “guardianship” of their mothers-in-law and husbands and have little say in household decisions and limited mobility outside the household. They would also be given the most menial household chores and eat last during meal times. The one refuge for *kanya*s would be their stay in their natal parents’ home, where they would receive greater care and have fewer duties. *Kanya*s would transition into their status as “guardians” when they became older, preferably a mother-in-law in their own right, but would also tend to acquire more privileges as younger brothers-in-law got married and brought in even younger wives to the household. Thus, it makes sense in our context to see older women, women with children or women staying in their natal home reporting greater agency.

Education and wealth followed a U-shape with small increases in education or wealth leading to increased pressure and only large increases leading to reduced pressure (Section 4.3). Previous reviews have found secondary education to be necessary for material changes to women’s household status (Jejeebhoy 1995; Pande, Malhotra, and Grown 2005) with smaller increases being predominantly associated with changes in social class. Evidence from studies in South Asia indicate lower female autonomy in middle-class families compared to lower class families due to stronger family status-related pressures (Cameron 1995; Liechty 1996; Maslak and Singhal 2008; Papanek 1979). As put in Bennett (1992, 15), “there is an inverse correlation between a woman’s status in the community (based on the economic status of her household […]) and her status within the household”. This contrasts with the findings in Vaz, Pratley, and Alkire (2016) in Chad who found no evidence for a relationship between education level and relative autonomy in their sample of women, while wealth was only correlated with autonomy in feeding infants, but not in other domains. The differences in findings between the Nepal and the Chad context point to the inadequacy of proxy measures such as education and wealth as global indicators women’s empowerment.

In our study, external pressure was positively correlated with internal motivation (Sections 4.1 and 4.3). This is consistent with work on the same scale in Chad (Vaz, Pratley, and Alkire 2016) which found either no evidence for correlation or positive correlations between identified regulation and external regulation and mostly positive correlations between identified regulation and introjected regulation across domains. In Bangladesh (Vaz et al. 2013), researchers using the same scale found external motivation to be strongly correlated with identified motivations as well. Multiple explanations may exist for why this correlation was found.

The first explanation may relate to the psychology of motivation itself. In addition to having first-order internal and external motivations for behaviour, there may be an underlying second-order factor for “general motivation” that measures whether participants are generally motivated in their activities. Particularly if the statements on internal motivation were strongly coloured by meanings of necessity, importance and survival (see Table 3) opposed to enjoyment or pleasure, then internal motivation may have been cognitively compatible with external pressure. Since a second-order structural model with an underlying factor predicting Factor E and Factor I is statistically equivalent to a model with a simple correlation between Factor E and Factor I, we cannot settle this question from statistical data alone, but must continue researching the psychosocial channels through which women experience autonomy to understand if such a factor exists.

A second explanation would be differential social desirability bias. A qualitative study on attitudes to wife beating found polar opposite attitudes expressed in survey and focus group data (Schuler and Islam 2008). Where survey results indicated that a high proportion of women found wife beating acceptable, women insisted in FGDs that they did not condone violence against women in the home and suggested harsh, sometimes graphic punishments for offenders. The researchers concluded that commonly used questions to measure attitudes to wife beating may be strongly biased by social norms in survey settings. In our context, women who were under greater external pressure from family members may simultaneously have been under greater pressure to report being motivated by interest or purpose in their daily activities, even if they did not feel so. However, conversations with local employees did not paint a unified picture as employees generally believed the social desirability of women’s agency to be an individual matter with the community neither strongly condemning nor strongly endorsing agency freedom in existing activities.

Finally, a causal relationship between external coercion and internal motivation may exist, a phenomenon sometimes referred to as “internalized oppression” (Rowlands 1997). National and international surveys also report higher rates of accepting attitudes towards wife beating among women who have experienced intimate partner violence compared to women who have not (García-Moreno et al. 2005; Kishor and Gupta 2004). The processes through which agents come to accept existing oppressive social norms and values as “natural” and “inevitable” to the extent that alternative social realities become practically inconceivable are important concepts in politics and sociology (Femia 1987; Freire 1972; Lukes 2005; Swartz 2012). In the Nepal context, women are often made to internalize ideas about their own inferiority since birth and expect to occupy the lowest rungs of the status hierarchy after marriage.

The last consideration poses questions regarding the match between empowerment and Sen’s agency freedom as measured by this survey. Sen and Alkire acknowledges the distorting effects of deprived environments on poor people’s wishes and desires in the concept of “adaptive preferences,” which forms the cornerstone of normative justifications for capability theory (Sen 1999). While they provide no guidance for how to account for adaptive preferences in data analysis (Ibrahim and Alkire 2007), Serene Khader’s “deliberative perfectionist” approach encourages us to adopt a minimal, vague and plural conception of flourishing based on cross-cultural deliberation as a basis for evaluating adaptive preferences. Although a useful starting point, she explicitly admits to not attempting to present a practicable methodology for large-scale evaluation of women’s empowerment (Khader 2011). One way to implement her deliberative approach in resource-constrained projects could be to complement quantitative surveys of empowerment with in-depth qualitative appraisals of carefully sampled groups or individuals.

For future uses of this tool, we recommend taking such a pragmatic approach. First, we recommend disaggregating the intervention effect into its component effects on external motivation E and internal motivation I (Table 4) and check if Factor I is affected. If Factor I is unaffected, adaptive preferences are not at issue since we have not registered a change in preferences. If, however Factor I is affected, we need to turn to in-depth qualitative work to evaluate the nature of the change in preferences observed according to our concept of basic flourishing arrived at through mutually respectful, cross-cultural deliberation.

## Notes

1. The distinction between “opportunity” and “exercise” freedom (Taylor 1985) mirrors the distinction between “dispositional” and “episodic” power (Haugaard 2010), while Amartya Sen’s concepts of “effective power” and “procedural control” (Sen 1985) also go under the names of “direct freedom” and “indirect freedom” (Pettit 2001).

2. The traditional approach in development economics to analyse such a situation would be to recast it as a bargaining problem between mother and child. The resultant portion allocations would be determined by each person’s relative bargaining power (Doss 2013). However, such an approach equates agency with bargaining power and assumes utility maximization as an axiom, two assumptions that are unhelpful in designing measures of freedom as they a priori make assumptions about constraints to the freedom of individuals to shape the relationship they live in and the freedom of individuals to follow or ignore their own happiness.

3. See also calls for “affiliative” dimensions of empowerment (Khader 2015).

4. The alignment is not perfect. With respect to its view on status-relationships, the Relative Autonomy Index could fall into either the Legitimacy or the Mastery categories depending on the interviewer and the interviewee's assumptions regarding the role of second-order choices, or choices about choices (Dworkin 1988). Suppose that a husband does not want his wife to take up employment and his wife adheres to him, because she has decided after a period of critical reflection that she wants to make him happy. If only her first-order choices are considered then her lack of employment is determined by her husband’s desires, i.e. it is externally motivated. However, if her second-order choices take priority, then her lack of employment is self-determined, since in this example, she has chosen not to choose, but to delegate her choice to her husband. From a Mastery perspective, she is potentially unfree, because she may be in position where it is difficult for her to make her own choices. From a Legitimacy perspective, she is potentially free, because she may feel that she is under a legitimate influence by her husband. The Relative Autonomy Index is ambiguous between these two perspectives.

5. Note, our tool differed from Vaz, Pratley, and Alkire (2016) in substituting the domain of major household purchases for health seeking behaviour.

6. Analyses were carried out in Mplus 7.11.

7. Data were scored using Stata/MP 13.

8. For women living in their husband’s home, “family” or *parivar* in Nepali and Maithili would be practically coterminous with “household” and refer to the group which “holds property jointly and observes most life-cycle and calendrical rituals together” (Bennett 1983). It would exclude neighbours, friends and blood or marital relatives living outside the household, except where these hold joint property rights such as husbands working as migrant labour. For women living in their natal home, *parivar* would refer to the natal household members living with them.

9. This change to our measure of agency might cause problems for contexts where women live alone as the only adult in their family as such women. In our context, only married women who were not widowed, divorced or separated were eligible to take part in the trial and so this issue would not arise.

10. A question arises as to whether the many modifications to the original tool produces a “new” tool—we believe that the changes in wording made to this tool captures the same theoretical construct as the “original” tool, namely agency freedom, but with better fit to the local context.

## About the Authors

**Lu Gram** is a Research Fellow at London School of Hygiene and Tropical Medicine and a Ph.D. student in Global Health at UCL. He has worked for years with the World Health Organization, DFID and Saving Newborn Lives across South Asia and Sub-Saharan Africa. His main research interest lies in the intersection between women’s empowerment, development economics and public health and the promotion gender equality through collective action in development programmes.

**Dr Joanna Morrison** is a social scientist at the University College London Institute for Global Health. She conducts research on health systems, and also in maternal and child health and nutrition, exploring how complex public health interventions change behaviour, and how culture affects systems, behaviours and uptake of care. She is interested in community mobilization for health improvement through participatory and action-oriented approaches. Most of her research is with partners in Nepal, but she is also working on projects in India, Bangladesh, Malawi and Niger.

**Neha Sharma** is head of qualitative research at MIRA (Mother Infant Research Activities) in Janakpur having worked as a researcher in large-scale randomized evaluations of participatory women’s groups for more than 5 years. Her research interests focus on women’s empowerment, participatory community-based approaches to public health, prevention and control of parasite infections and intersections between public health and veterinary science.

**Bhim Shrestha** is a senior research coordinator at MIRA and has had more than 10 years of experience in implementing and monitoring large-scale evaluations of participatory women’s groups across a range of districts in Nepal. He is particularly interested in issues of implementation science, management and evaluation of complex interventions and participatory action-based approaches to maternal and child health.

**Dharma Manandhar**, President and Executive Director of MIRA (Mother and Infant Research Activities), an NGO, has been involved in hospital- and community-based studies on maternal and infant health for over two decades and has received many national awards. Besides being the Founder President of Perinatal Society of Nepal, a Past President of Nepal Pediatric Society and a Fellow of several professional bodies in Nepal, India and UK, he has been involved in many committees on maternal and child health, has designed simple low-cost equipment, authored books on newborn care, published many articles and presented in many conferences.

**Anthony Costello** is the Director at UCL Institute for Global Health and Centre for International Health and Development. He has been at the cutting edge of developing low-cost interventions to improve maternal and child health in Southern Asia and Africa, particularly work using peer education amongst women’s groups to demonstrate through randomized controlled trials the ability of such interventions to reduce newborn mortality. Through the Institute for Global Health, he has brought a multi-disciplinary approach to complex health problems. A major recent achievement is his Chairmanship of the UCL/Lancet Commission on Managing the Health Effects of Climate Change, published in the Lancet in May, 2009. This has been influential to national and international policy makers.

**Naomi Saville** is a Senior Research Associate at UCL Institute for Global Health. She is based in Nepal where she conducts cluster-randomized trials and other research associated with maternal and child nutrition and health. Since 2005 Naomi has been testing the impact of participatory learning and action women’s groups on health outcomes, in particular neonatal mortality, birth weight and maternal/child nutrition. Other research interests include prevention of stunting and health inequalities. Previously Naomi trained in zoology at the University of Cambridge and worked on livelihood diversification and community development in Nepal, India, Somalia, Sierra Leone, and Trinidad and Tobago.

**Dr Jolene Skordis-Worrall** is an Economist specializing in Applied Health and Development Economics. With strong networks in sub-Saharan Africa and South East Asia, she has experience of working with a wide range of international organizations. She works primarily within randomized trials of complex public health interventions, establishing research programmes that span gender empowerment and capability, multidimensional poverty and antipoverty transfer programmes, social networks, health equity and understanding fiscal space for sustainable and scalable programme delivery. She has published widely in high impact journals and has a strong international reputation. Her work has directly influenced government policy.
